# Supplementary material for: Clinical characteristics and outcomes of intraocular lens dislocation: an eight-year retrospective study
Source: PLoS One. 2025 Jun 25;20(6):e0327210. doi: 10.1371/journal.pone.0327210 (PMC12193011; doi:10.1371/journal.pone.0327210)
Supplement: S1 File — (PDF) [file pone.0327210.s001.pdf]

ID	性别	诱因	间隔	间隔	症状	烟	酒	高	糖	第一次情况	年龄	眼轴	手术方式	UDVA1	UDVA1logmar	UDVA2	UDVA2logmar	ΔUDVA	IOP	并发症
P1	女		2	2				1		白内障	52	23.01	0	0.01	2.00					
P2	男		1月	0.083		6				外伤	32	23.49	0	0.02	1.70					
P3	男		10	10						白内障	59	20.60	0	LP	4.00					
P4	男		3	3				1		白内障	52	23.52	0	0.16	0.80					
P5	男		7	7						白内障	49	26.92	0	0.2	0.70					
P6	女		12	12						白内障	67	33.6	0	0.3	0.52					
P7	男		15	15						白内障	57	26.2	0	0.32	0.49					
P8	男		15	15						先天障	24	24.35	0	0.25	0.60					
P9	女		25	25						白内障	41	23.9	0	1	0.00					
P10	男		14	14						白内障	61	33.24	0	HM	3.00					
P11	男		7	7						白内障	51	23.28	0	0.25	0.60					
P12	女		8	8						脱位	15	26.3	0	0.8	0.10					
P13	女		2	2						外伤	8	23.09	1	0.16	0.80	0.2	0.70	0.10	13	
P14	女		3周	0.058	遮挡感					白内障	66	22.57	1	0.8	0.10	0.32	0.49	-0.40	18	
P15	女		9	9						脱位	44	21.56	1	0.2	0.70	0.5	0.30	0.40	27	眼压高：溴莫尼定
P16	女		5	5	重影					白内障	7	25.78	1	0.03	1.52	0.12	0.92	0.60	9	
P17	女		14	14						脱位	24	26.32	1	0.5	0.30	0.8	0.10	0.20	16	
P18	女		7	7	痛					外伤	63	23.07	1	LP	4.00	0.25	0.60	3.40	7	
P19	女		8	8	黑影					白内障	44	26.35	1	HM	3.00	0.06	1.22	1.78	16	
P20	女		3	3				1		白内障	68	23.88	1	0.1	1.00	0.03	1.52	-0.52	15	
P21	女	搬运重物	4	4	重影			1	1	白内障	51	22.20	1	0.1	1.00	0.32	0.49	0.51	15	
P22	女	塑料玩具砸伤	12	12						先天障	27	22.37	1	0.7	0.15	0.12	0.92	-0.77	21	
P23	女		8	8						白内障	64	23.70	1	HM	3.00	HM	3.00	0.00	7	
P24	男		8	8	异物感					脱位	22	25.57	1	0.4	0.40	0.8	0.10	0.30	13	
P25	男	揉眼后	4	4						白内障	59	24.85	1	0.32	0.49	1.0	0.00	0.49	19	
P26	男	羽毛球打伤	3	3	红	30	30			白内障	67	24.59	1	0.12	0.92	0.8	0.10	0.82	16	
P27	男	搬运重物	5	5						外伤	61	22.64	1	0.32	0.49	0.32	0.49	0.00	20	
P28	男		4	4						白内障	41	24.38	1	1.0	0.00	0.8	0.10	-0.10	34	眼压高：前房穿刺
P29	男		1天	0.003				1		白内障	66	23.69	1	0.25	0.60	0.8	0.10	0.51	10	
P30	男	插销弹伤	13	13	红痛	20		1		白内障	36	26.19	1	CF	2.00	0.06	1.22	0.78	7	
P31	男		1	1	斜视					白内障	6	21.07	1	0.1	1.00	0.05	1.30	-0.30	16	
P32	男		8	8						白内障	73	23.97	1	0.01	2.00	0.4	0.40	1.60	11	
P33	男		7	7						外伤	13	23.06	1	HM	3.00	CF	2.00	1.00	13	
P34	男		4	4				1		白内障	55	23.04	1	0.2	0.70	0.63	0.20	0.50	14	
P35	男	碰伤后	10	10				1		脱位	72	25.09	1	0.6	0.22	0.63	0.20	0.02	16	
P36	男		1月	0.083						白内障	30	29.17	1	CF	2.00	CF	2.00	0.00	7	
P37	男	揉眼后	8	8		40	40	1		白内障	59	23.59	1	CF	2.00	0.5	0.30	1.70	17	
P38	男		5	5						白内障	75	24.13	1	0.32	0.49	0.4	0.40	0.10	19	
P39	男		13日	0.036		20				白内障	58	22.57	1	0.5	0.30	0.5	0.30	0.00	16	
P40	男		3月	0.25	重影					白内障	49	31.08	1	0.25	0.60	0.25	0.60	0.00	16	
P41	男		1天	0.003	痛、呕吐					白内障	67	23.85	1	CF	2.00	CF	2.00	0.00	14	
P42	男		1	1						脱位	57	26.02	1	0.8	0.10	0.8	0.10	0.00	21	
P43	男		23	23	复视	20				外伤	34	26.41	1	0.2	0.70	0.2	0.70	0.00	7	
P44	男		4月	0.333						外伤	51	23.44	1	0.01	2.00	0.4	0.40	1.60	11	
P45	男		3	3						白内障	59	26.23	1	0.12	0.92	0.25	0.60	0.32	16	
P46	男		2	2		20		1		白内障	61	23.98	1	0.2	0.70	0.2	0.70	0.00	9	
P47	男		5	5	胀痛			1		白内障	55	27.57	1	0.04	1.40	CF	2.00	-0.60	19	
P48	男		17	17	复查发现，无感	1				先天障	18	24.22	1	0.12	0.92	0.2	0.70	0.22	30	眼压高：布林佐胺，溴莫尼定
P49	男		5	5						脱位	57	23.80	1	0.12	0.92	0.63	0.20	0.72	8	
P50	男		4	4		40		1		白内障	72	25.27	1	0.25	0.60	0.4	0.40	0.20	20	
P51	男	外伤	1天	0.003	角膜水肿					白内障	74	23.76	1	0.08	1.10	0.12	0.92	0.18	13	
P52	男		7	7						白内障	16	25.26	1	0.05	1.30	0.32	0.49	0.81	7	
P53	男		2	2				1	1	白内障	54	24.12	1	0.2	0.70	0.8	0.10	0.60	15	
P54	男	书本敲打	9	9						外伤	13	26.04	1	0.03	1.52	0.04	1.40	0.12	17	
P55	男		8	8	痛	35		1	1	白内障	63	23.21	1	0.6	0.22	0.8	0.10	0.12	21	
P56	男	情绪激动	3	3					1	白内障	59	23.71	1	0.63	0.20	0.05	1.30	-1.10	18	
P57	男		10	10		50		1		白内障	73	24.46	1	0.1	1.00	0.25	0.60	0.40	12	
P58	男		5天	0.014	重影，痛			1		脱位	23	24.37	1	0.6	0.22	0.8	0.10	0.12	14	
P59	男		15日	0.041		17	10			白内障	29	27.29	1	0.02	1.70	0.4	0.40	1.30	21	
P60	男		8	8						外伤	57	26.86	1	0.3	0.52	0.3	0.52	0.00	16	

P61	男		3	3						先天障	6	25.26	1	0.16	0.80	1.0	0.00	0.80	15	
P62	男		10	10						白内障	82	24.38	1	0.2	0.70	0.32	0.49	0.20	14	
P63	男	抬头后	1	1				1		脱位+白内障	55	26.23	1	0.1	1.00	0.08	1.10	-0.10	14	
P64	男		9	9						白内障	19	22.39	1	CF	2.00	0.2	0.70	1.30	10	
P65	男		1	1				1	1	白内障	65	24.50	1	0.8	0.10	0.4	0.40	-0.30	20	
P66	男		20	20		30				白内障	56	30.94	1	0.05	1.30	0.1	1.00	0.30	11	
P67	男		4	4			30	1		白内障	51	22.94	1	0.01	2.00	0.8	0.10	1.90	18	
P68	女		8	8						脱位	15	25.78	1	0.25	0.60	0.5	0.30	0.30	9	
P69	女	木柴打伤	8	8	红痛					白内障	60	25.70	1（非悬吊）	0.4	0.40	1.0	0.00	0.40	11	
P70	女		3	3	重影，畏光					外伤	37	25.05	1（非悬吊）	0.4	0.40	0.4	0.40	0.00	15	
P71	女	摔倒磕伤	20	20						白内障	58	33.03	1（非悬吊）	0.01	2.00	0.03	1.52	0.48	13	
P72	女		10	10		20				白内障	87	23.67	1（非悬吊）	LP	4.00	LP	4.00	0.00	16	
P73	女	手肘撞击	6	6	重影，红、痛					白内障	41	29.75	1（非悬吊）	0.4	0.40	0.4	0.40	0.00	13	
P74	女	硬物打伤	8	8				1		白内障	65	28.16	1（非悬吊）	0.04	1.40	0.12	0.92	0.48	17	
P75	男		11	11	异物感	15				白内障	61	34.70	1（非悬吊）	0.2	0.70	0.32	0.49	0.20	37	眼压高：前房穿刺
P76	男		3	3	复查发现，无感					白内障	7	21.26	1（非悬吊）	0.3	0.52	0.2	0.70	-0.18	16	
P77	男		24	24	红、胀					外伤	26	27.60	1（非悬吊）	0.01	2.00	0.01	2.00	0.00	11	
P78	男	揉眼后	2	2	干涩			1		外伤	54	24.24	1（非悬吊）	0.01	2.00	0.02	1.70	0.30	19	
P79	男	碰伤	18	18	黑影			1		白内障	68	25.79	1（非悬吊）	0.01	2.00	0.5	0.30	1.70	16	
P80	男		17	17						先天障	24	23.93	1（非悬吊）	0.06	1.22	0.5	0.30	0.92	21	
P81	男		15	15	痛涩红					先天障	24	24	1（非悬吊）	0.32	0.49	0.16	0.80	-0.30	8	
P82	男		1月	0.083	复视					外伤	32	27.99	1（未悬吊）	1.0	0.00	1.0	0.00	0.00	16	
P83	女		2	2						白内障	61	30.70	1+玻切	0.25	0.60	0.4	0.40	0.20	21	
P84	女		10	10						外伤	19	24.87	1+玻切	0.1	1.00	0.2	0.70	0.30	13	
P85	男		3天	0.044						外伤	61	24.91	1+玻切	CF	2.00	0.25	0.60	1.40	15	
P86	男		2	2						白内障	51	24.22	1+玻切	CF	2.00	0.05	1.30	0.70	29	眼压高：溴莫尼定，布林佐胺
P87	男		1	1		50		1		白内障	64	25.27	1+玻切	0.02	1.70	0.05	1.30	0.40	13	
P88	男		1	1						白内障	54	23.72	1+玻切	0.6	0.22	0.5	0.30	-0.08	21	
P89	男		1	1						白内障	54	23.33	1+玻切	0.4	0.40	0.63	0.20	0.20	20	
P90	男		10	10		45				白内障	60	25.22	1+玻切（非悬吊）	0.2	0.70	0.8	0.10	0.60	9	
P91	女		14	14				1	1	白内障	66	22.7	1+经结膜玻切	0.05	1.30	0.12	0.92	0.38	16	
P92	女		10	10	重影					脱位	23	29.45	1+经结膜玻切	0.05	1.30	0.1	1.00	0.30	14	
P93	男	揉眼后	8	8				1		白内障	74	23.44	1+囊膜撕除+前段玻	0.16	0.80	0.16	0.80	0.00	14	
P94	男	揉眼后	4	4						白内障	67	23.52	1+囊膜撕除+前段玻	0.25	0.60	CF	2.00	-1.40	13	
P95	女		6	6				1		白内障	50	25.31	1+前段玻切	0.63	0.20	1.0	0.00	0.20	13	
P96	女		6	6				1		白内障	72	24.10	1+前段玻切	0.02	1.70	0.1	1.00	0.70	33	眼压高：溴莫尼定
P97	男	揉眼后	1	1				1		白内障	51	23.15	1+前段玻切	0.63	0.20	0.63	0.20	0.00	11	
P98	男		4	4						白内障	49	37.90	1+前段玻切	0.06	1.22	0.12	0.92	0.30	9	
P99	男		2	2	红涩	20				白内障	39	24.08	1+前段玻切	0.04	1.40	0.02	1.70	-0.30	38	眼压高：甘露醇
P100	男		15	15						白内障	66	23.74	1+前段玻切	0.4	0.40	0.8	0.10	0.30	11	
P101	男		3	3		30	30			白内障	58	24.19	1+前段玻切	0.25	0.60	0.01	2.00	-1.40	30	眼压高：溴莫尼定
P102	男		25	25		30				白内障	55	24.31	1+前段玻切	0.25	0.60	0.63	0.20	0.40	18	
P103	男		4	4		10	10		1	白内障	57	24.42	1+前段玻切	0.63	0.20	0.8	0.10	0.10	15	
P104	男		2	2		30				白内障	49	23.53	1+前段玻切	0.4	0.40	1.0	0.00	0.40	10	
P105	男		6	6	视物变形，红					白内障	64	22.50	1+前段玻切	0.4	0.40	0.2	0.70	-0.30	14	
P106	男		7	7				1	1	白内障	61	23.82	1+前段玻切	1.0	0.00	0.12	0.92	-0.92	19	
P107	男	木棒击打	1	1						白内障	60	22.90	1+前段玻切	0.2	0.70	0.4	0.40	0.30	15	
P108	男	揉眼后	15	15		30				白内障	57	25.86	1+前段玻切	0.06	1.22	0.8	0.10	1.12	16	
P109	女		25	25						白内障	41	24.99	1+前段玻切	0.8	0.10	0.5	0.30	-0.20	15	
P110	男		6月	0.5		20		1		白内障	69	24.14	1+前段玻切	0.1	1.00	0.2	0.70	0.30	35	眼压高：前房穿刺
P111	男		3	3		30				白内障	55	29.69	1	0.2	0.70	0.4	0.40	0.30	21	
P112	男	车祸摔伤	6	6	红痛			1	1	白内障	60	22.49	1	CF	2.00	0.1	1.00	1.00	24	眼压高：溴莫尼定
P113	女		5	5						先天障	8	24.16	2	0.08	1.10	0.1	1.00	0.10	30	眼压高：前房穿刺
P114	女		10	10						脱位	13	28.78	2	0.2	0.70	0.3	0.52	0.18	11	
P115	女		12	12						外伤	38	23.07	2	CF	2.00	HM	3.00	-1.00	7	
P116	女		14	14						白内障	55	23.75	2	0.6	0.22	0.5	0.30	-0.08	9	
P117	女		1	1						白内障	65	30.26	2	0.2	0.70	0.05	1.30	-0.60	19	
P118	男	篮球碰伤	7	7						脱位	16	26.63	2	0.32	0.49	0.8	0.10	0.40	31	眼压高：前房穿刺
P119	男		4	4		30				白内障	46	25.22	2	0.4	0.40	0.6	0.22	0.18	12	
P120	男		8	8						白内障	49	22.38	2	CF	2.00	CF	2.00	0.00	13	
P121	男		4	4		30				白内障	59	24.93	2	0.02	1.70	0.8	0.10	1.60	7	
P122	男		7	7		10		1		白内障	57	26.30	2	0.63	0.20	0.1	1.00	-0.80	30	眼压高：前房穿刺

P123	男		17	17						白内障	69	23.16	2	0.7	0.15	0.25	0.60	-0.45	12	
P124	男		15	15						白内障	65	25.23	2	0.05	1.30	0.2	0.70	0.60	11	
P125	男		9	9						先天障	21	28.3	2	0.07	1.15	0.16	0.80	0.36	20	
P126	男		15	15						白内障	68	25.3	2	0.02	1.70	0.2	0.70	1.00	11	
P127	女		0.5	0.5	痛			1		白内障	59	23.38	2（非悬吊）	0.08	1.10	0.32	0.49	0.60	16	
P128	男		24	24						外伤	30	25.11	2（非悬吊）	0.05	1.30	0.12	0.92	0.38	10	
P129	男		17	17						先天障	24	23.7	2（非悬吊）	0.16	0.80	0.25	0.60	0.19	17	
P130	女		6	6						白内障	53	24.33	2+玻切	0.03	1.52	0.4	0.40	1.12	15	
P131	女		8	8						白内障	54	22.28	2+玻切	CF	2.00	0.2	0.70	1.30	13	
P132	女		25	25						外伤	29	28.29	2+玻切	0.2	0.70	0.32	0.49	0.20	21	眼压高：布林唑胺
P133	女		1日	0.003				1		白内障	77	22.87	2+玻切	0.08	1.10	0.12	0.92	0.18	13	
P134	男		14	14				1		白内障	63	24.51	2+玻切	0.05	1.30	0.3	0.52	0.78	22	
P135	男		8	8						白内障	60	30.63	2+玻切	CF	2.00	CF	2.00	0.00	49	眼压高：前房穿刺
P136	男		2月	0.167		50	50	1	1	白内障	74	22.40	2+玻切	0.12	0.92	0.5	0.30	0.62	15	
P137	男		13	13	视物变形					先天障	21	28.53	2+玻切	0.12	0.92	0.1	1.00	-0.08	17	
P138	男		13	13				1	1	白内障	68	23.45	2+玻切	0.04	1.40	0.63	0.20	1.20	32	眼压高：前房穿刺
P139	男		19	19						脱位	37	28.92	2+玻切	0.4	0.40	0.5	0.30	0.10	7	
P140	男		10	10	痛					白内障	22	25.09	2+囊膜切除	0.05	1.30	0.12	0.92	0.38	21	
P141	女		6	6				1		白内障	64	23.38	2+前段玻切	0.5	0.30	0.02	1.70	-1.40	44	眼压高：噻吗洛尔
P142	男		14	14		40				白内障	67	26.97	2+前段玻切	0.5	0.30	0.2	0.70	-0.40	38	眼压高：甘露醇
P143	男		3	3		30				白内障	49	23.22	2+前段玻切	CF	2.00	0.8	0.10	1.90	18	
P144	男		6	6					1	白内障	53	22.91	2+前段玻切	0.4	0.40	0.8	0.10	0.30	19	
P145	男		17	17	胀痛、红					白内障	54	22.65	2+前段玻切	0.8	0.10	0.25	0.60	-0.51	10	
P146	男		5	5	重影					白内障	57	24.07	2+前段玻切	0.1	1.00	0.16	0.80	0.20	16	
P147	男		2	2						白内障	60	24.47	2+前段玻切	0.1	1.00	0.05	1.30	-0.30	11	
P148	男		10	10				1		白内障	72	22.81	2+前段玻切	0.02	1.70	0.02	1.70	0.00	20	
P149	男		6	6						白内障	48	25.52	2+前段玻切	0.01	2.00	0.5	0.30	1.70	17	
P150	男		16	16						先天障	21	27.28	2+前段玻切	0.16	0.80	0.25	0.60	0.19	7	
P151	男		2	2						白内障	6	28.56	2+前段玻切（非悬吊）	0.5	0.30	0.5	0.30	0.00	13	
P152	女		1	1						白内障	56	32.65	3	0.04	1.40	0.12	0.92	0.48	10	
P153	女	摔倒磕伤	6	6				1	1	白内障	67	28	3	0.2	0.70	0.2	0.70	0.00	21	
P154	男		11天	0.030	复查发现，无感			1		白内障	45	32.59	3	0.1	1.00	0.1	1.00	0.00	16	
P155	男		2月	0.167	合并角膜水肿					外伤	40	24.90	3	HM	3.00	HM	3.00	0.00	16	
P156	男		15	15	角膜缘变性，房闪，KP					白内障	18	22.43	3	LP	4.00	HM	3.00	1.00	7	
P157	女		12	12						白内障	67	34.06	3	0.1	1.00	0.2	0.70	0.30	21	
P158	男		14	14					1	白内障	61	32.72	3	0.12	0.92	0.2	0.70	0.22	20	
P159	男		18	18	遮挡感					白内障	73	25.96	3+玻切	0.08	1.10	0.07	1.15	-0.06	12	
P160	男		14	14		3				白内障	30	25.05	3+玻切	CF	2.00	CF	2.00	0.00	10	
P161	男		1周	0.019						白内障	45	24.73	3+玻切	CF	2.00	0.01	2.00	0.00	15	
P162	男		23	23	复查发现，无感					白内障	27	29.89	3+前段玻切	CF	2.00	CF	2.00	0.00	10	
P163	男		1	1						白内障	59	23.87	3	0.05	1.30	0.01	2.00	-0.70	17	
P164	女		2	2				1		白内障	65	22.33	囊膜切除	0.4	0.40	0.63	0.20	0.20	18	
P165	男		1	1						白内障	63	22.34	囊膜切除	0.4	0.40	0.12	0.92	-0.52	17	
P166	男		4	4				1	1	白内障	70	24.20	囊膜切除	0.04	1.40	0.25	0.60	0.80	13	

group1 **rank sum test**

描述统计

	N	平均值	标准差	最小值	最大值	百分位数 第 25 个	第 50 个（中位数）	第 75 个
UDVA	200	0.8750	0.77413	0.00	4.00	0.3000	0.7000	1.2800
group	200	1.5000	0.50125	1.00	2.00	1.0000	1.5000	2.0000

秩

group		N	秩平均值	秩的总和
UDVA	1.00	100	113.51	11351.00
	2.00	100	87.49	8749.00
	总计	200		

检验统计[a]

	UDVA
曼-惠特尼 U	3699.000
威尔科克森 W	8749.000
Z	-3.187
渐近显著性（双尾）	0.001

a. 分组变量：group

group2 **rank sum test**

描述统计

	N	平均值	标准差	最小值	最大值	百分位数 第 25 个	第 50 个（中位数）	第 75 个
UDVA	78	0.9073	0.62559	0.10	3.00	0.4000	0.8000	1.3000
group	78	1.5000	0.50324	1.00	2.00	1.0000	1.5000	2.0000

秩

group		N	秩平均值	秩的总和
UDVA	1.00	39	45.03	1756.00
	2.00	39	33.97	1325.00
	总计	78		

检验统计[a]

	UDVA
曼-惠特尼 U	545.000

威尔科克森 W	1325.000
Z	-2.159
渐近显著性（双尾）	0.031

a. 分组变量：group

group3 rank sum test

描述统计

	N	平均值	标准差	最小值	最大值	百分位数 第 25 个	百分位数 第 50 个（中位数）	百分位数 第 75 个
UDVA	24	1.6496	0.89754	0.70	4.00	0.9400	1.3500	2.0000
group	24	1.5000	0.51075	1.00	2.00	1.0000	1.5000	2.0000

秩

	group	N	秩平均值	秩的总和
UDVA	1.00	12	13.00	156.00
	2.00	12	12.00	144.00
	总计	24		

检验统计[a]

	UDVA
曼-惠特尼 U	66.000
威尔科克森 W	144.000
Z	-0.352
渐近显著性（双尾）	0.725
精确显著性[2*(单尾显著性)]	.755[b]

a. 分组变量：group

b. 未针对绑定值进行修正。

Complications Chi-square test

个案处理摘要

	个案 有效 N	有效 百分比	缺失 N	缺失 百分比	总计 N	总计 百分比
组别 * 发生	139	100.0%	0	0.0%	139	100.0%

组别 * 发生 交叉表

			发生		
			无	有	总计
组别	1.00	计数	90	10	100
		期望计数	87.1	12.9	100.0
		占 组别 的百分比	90.0%	10.0%	100.0%
		占 发生 的百分比	74.4%	55.6%	71.9%
	2.00	计数	31	8	39
		期望计数	33.9	5.1	39.0
		占 组别 的百分比	79.5%	20.5%	100.0%
		占 发生 的百分比	25.6%	44.4%	28.1%
总计		计数	121	18	139
		期望计数	121.0	18.0	139.0
		占 组别 的百分比	87.1%	12.9%	100.0%
		占 发生 的百分比	100.0%	100.0%	100.0%

卡方检验

	值	自由度	渐进显著性（双侧）	精确显著性（双侧）	精确显著性（单侧）
皮尔逊卡方	2.751[a]	1	0.097		
连续性修正[b]	1.897	1	0.168		
似然比	2.553	1	0.110		
费希尔精确检验				0.157	0.087
线性关联	2.731	1	0.098		
有效个案数	139				

a. 0 个单元格 (0.0%) 的期望计数小于 5。最小期望计数为 5.05。

b. 仅针对 2x2 表进行计算
